# Supplementary material for: PTEN-AKT2 regulates mixed lineage liver cancer development and sensitizes cancer cells to TGFβ treatment
Source: Oncogene. 2026 Aug 20;45(41):4362–76. doi: 10.1038/s41388-026-03960-0 (PMC13645676; doi:10.1038/s41388-026-03960-0)
Supplement: Supplementary file 1 [file 41388_2026_3960_MOESM1_ESM.pdf]

Supplementary Methods

siRNA and Plasmid Transfection

Silencer® Select siRNA targeting SOX9 (Thermo Fisher Scientific, Waltham, MA, USA, AM16708) and a non-targeting control siRNA (Thermo Fisher Scientific, AM4611) were transfected using Lipofectamine RNAiMAX (Thermo Fisher Scientific, #13778075) according to the manufacturer's instructions. shSOX9 plasmid (VectorBuilder, VB900123-7988yjz), NICD1 expression plasmid (Addgene, #20183), and corresponding empty vector controls were introduced using Lipofectamine 3000 (Thermo Fisher Scientific, #L3000008) following the manufacturer's protocol.

Antibody used for immunoblotting and immunohistochemistry.

Antibody	Catalog #	Vendor	Research Resource Identifiers
Activated Notch1	ab8925	Abcam	RRID: AB_306863
Akt (pan)	4691	Cell Signaling Technology	RRID: AB_915783
AKT1	2938	Cell Signaling Technology	RRID: AB_915788
AKT2	3063	Cell Signaling Technology	RRID: AB_2225186
Arginase-1	93668	Cell Signaling Technology	RRID: AB_2800207
Beta actin	A5441	Sigma	RRID: AB_476744
Beta actin	66009-1-Ig	Proteintech	RRID: AB_2687938
Cleaved Notch1 (Val1744)	4147	Cell Signaling Technology	RRID: AB_2153348
EPCAM	21050-1-AP	Proteintech	RRID: AB_10693684
GAPDH	G9545	Sigma	RRID: AB_796208
HepPar 1	M7158	Dako	RRID: AB_3720362
Hes1	11988	Cell Signaling Technology	RRID: AB_2728766
HNF-4-alpha	ab41898	Abcam	RRID: AB_732976
Jagged1	sc-390177	Santa Cruz Biotechnology	RRID: AB_2892141
pan-AKT1/2/3	sc-8312	Santa Cruz Biotechnology	RRID: AB_671714
Pan-CK	Z0622	Dako	RRID: AB_2650434
phospho-AKT (Ser473)	4060	Cell Signaling Technology	RRID: AB_231504
phospho-AKT (Thr308)	9275	Cell Signaling Technology	RRID: AB_329828
phospho-PRAS40 (Thr246)	2640	Cell Signaling Technology	RRID: AB_916141
phospho-SMAD3 (Ser423/425)	9520	Cell Signaling Technology	RRID: AB_2193207
Pten	9559	Cell Signaling Technology	RRID: AB_2253290
SMAD3	9523	Cell signaling Technology	RRID: AB_2193182
SMAD4 (B-8)	sc-7966	Santa Cruz Biotechnology	RRID: AB_627905
SOX9	AMAB90795	Sigma	RRID: AB_2665670
SOX9	82630	Cell Signaling Technology	RRID: AB_2665492
Vinculin	26520-1-AP	Proteintech	RRID: AB_2868558
YFP	ab6673	Abcam	RRID: AB_305643

Primers used for quantitative PCR.

**Primers**	**Sequence 5'-3'**
Human SOX9 F	CCAGAACAAGCCGCACGTCA
Human SOX9 R	CTGCCCCGTTCTTCACCGACT
Human EpCAM F	GCGAGTGAGAACCTACTGGA
Human EpCAM R	CGCGTTGTGATCTCCTTCTG
Human PROM1 F	AAGGCATATGAATCCAAAATTGA
Human PROM1 R	CCACCAGAGGCATCAGAATAA
Human GapDH F	AGGGCTGCTTTTAACTCTGGT
Human GapDH R	CCCCACTTGATTTTGGAGGGA
Mouse Sox9 F	GTGCAAGCTGGCAAAGTTGA
Mouse Sox9 R	TGCTCAGTTCACCGATGTCC
Mouse Hes1 F	ACACCGGACAAACCAAAGAC
Mouse Hes1 R	AATGCCGGGAGCTATCTTTC
Mouse Notch1 F	TGTGGCTTCCTTCTACTGCG
Mouse Notch1 R	CTTTGCCGTTGACAGGGTTG
Mouse Ccn2 F	CAGCGGTGAGTCCTTCCAAA
Mouse Ccn2 R	TCTTCCAGTCGGTAGGCAGC
Mouse Serpine1 F	ACTTCACAAGTCTTTCCGACCA
Mouse Serpine1 R	ACTGTGCCGCTCTCGTTTAC
Mouse Epcam F	ATCGCTGTCATTGTGGTGGT
Mouse Epcam R	ACCCATCTCCTTTATCTCAGCC
Mouse Prom1 F	CTGGACCGGAGAGGGATGGT
Mouse Prom1 R	TCCACACAGCCCCAGTAACA
Mouse 18s F	CGGCTACCACATCCAAGGAA
Mouse 18s R	GCTGGAATTACCGCGGCT
