# Supplementary material for: PTEN-AKT2 regulates mixed lineage liver cancer development and sensitizes cancer cells to TGFβ treatment
Source: Oncogene. 2026 Aug 20;45(41):4362–76. doi: 10.1038/s41388-026-03960-0 (PMC13645676; doi:10.1038/s41388-026-03960-0)
Supplement: Supplementary file 2 [file 41388_2026_3960_MOESM2_ESM.pdf]

**A**

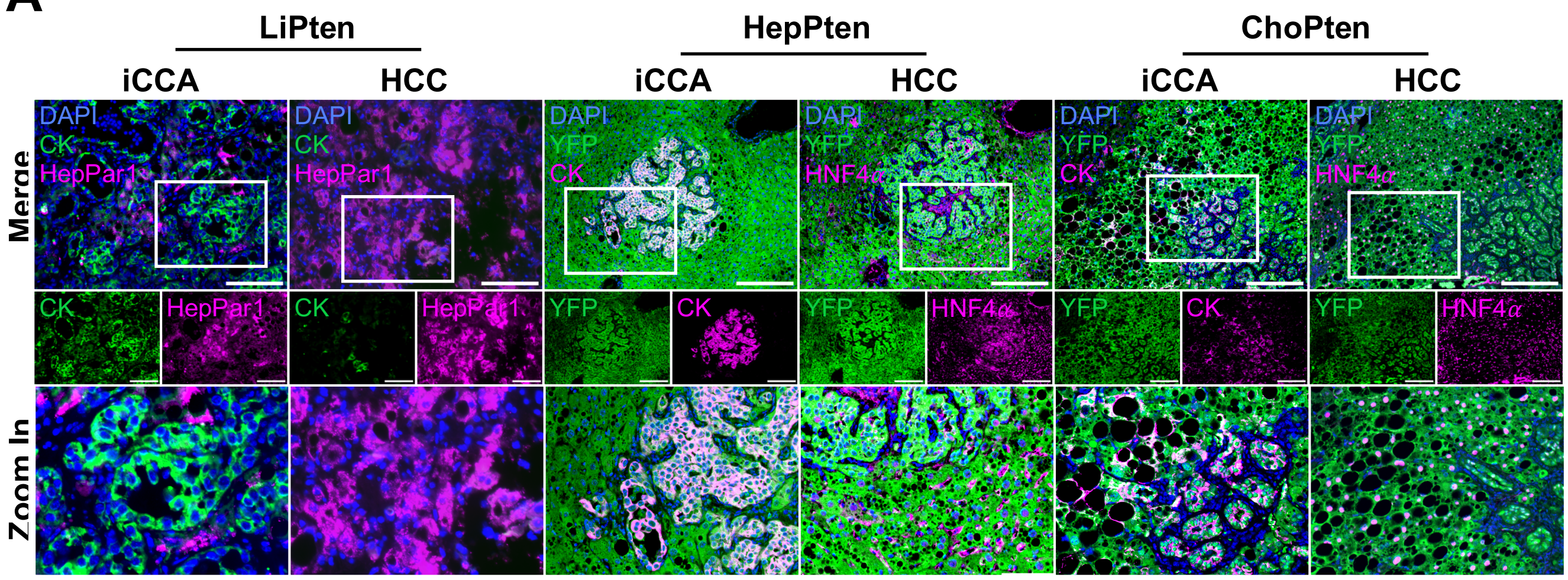


**B**

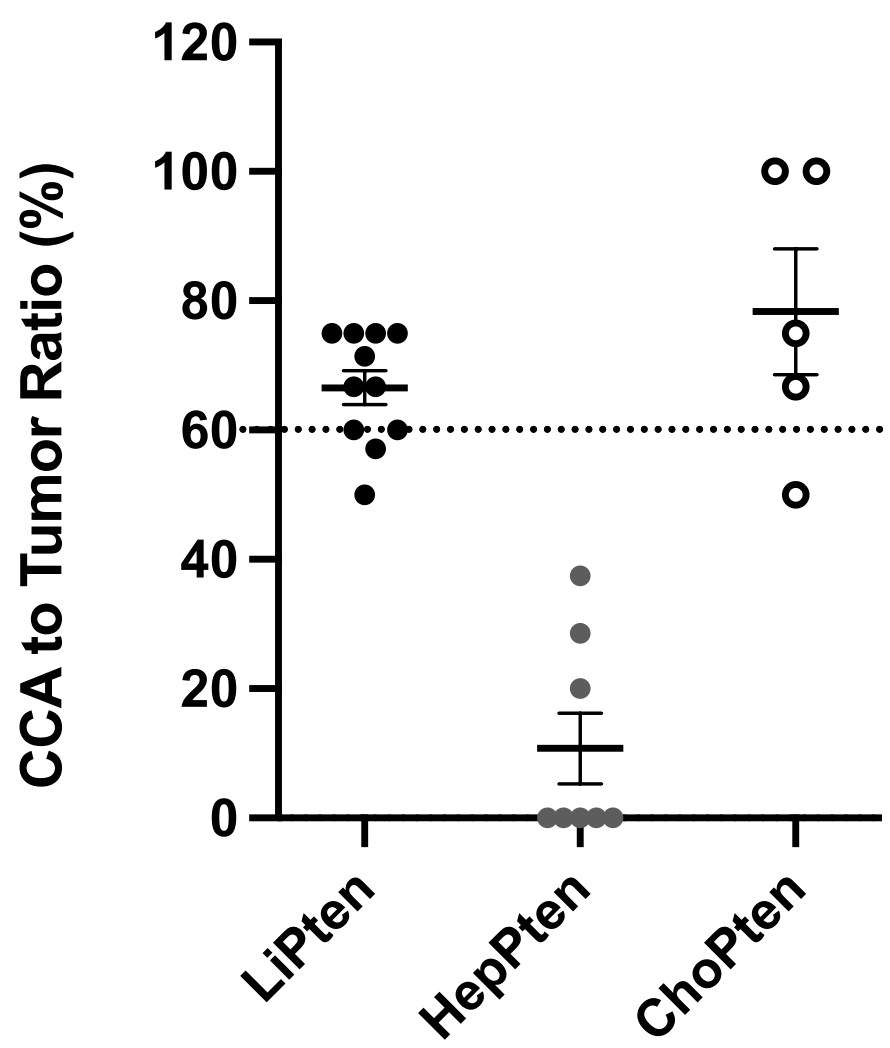


**Supplemental Figure 1. PTEN loss in liver parenchymal leads to CCA-dominant heterogeneous tumors. (A)** Immunofluorescent co-staining of HepPar-1 and CK in LiPten livers, and co-staining of YFP with HNF4a or CK in HepPten and ChoPten livers to identify HCC and iCCA areas, respectively. The tissues are co-stained with DAPI (blue) to show nuclei. Scale bar 50 µm. **(B)** Quantification of CCA development in mouse models lacking *Pten* in the liver. Percentage of the tumors that are CCA vs. total tumors developed in each mouse. Each dot represents a mouse.

**A**

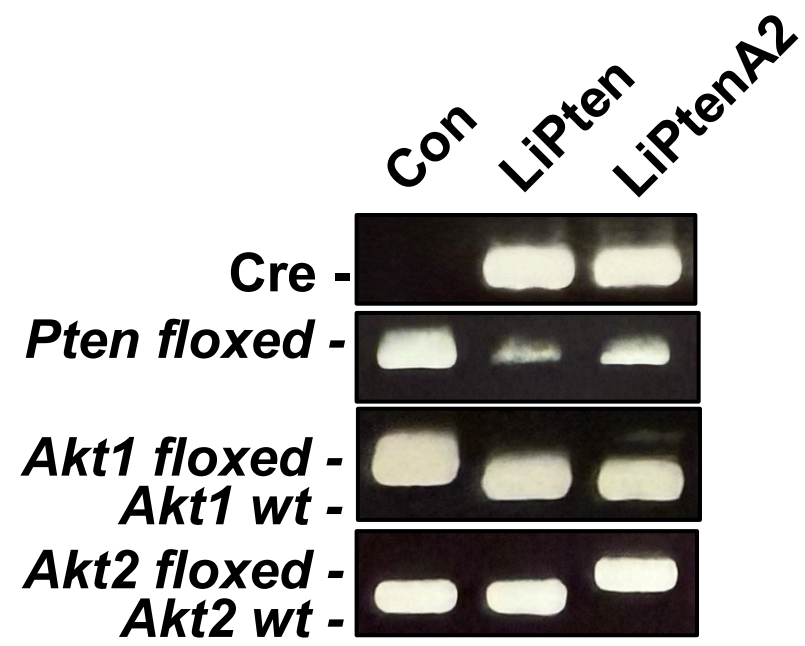


**B**

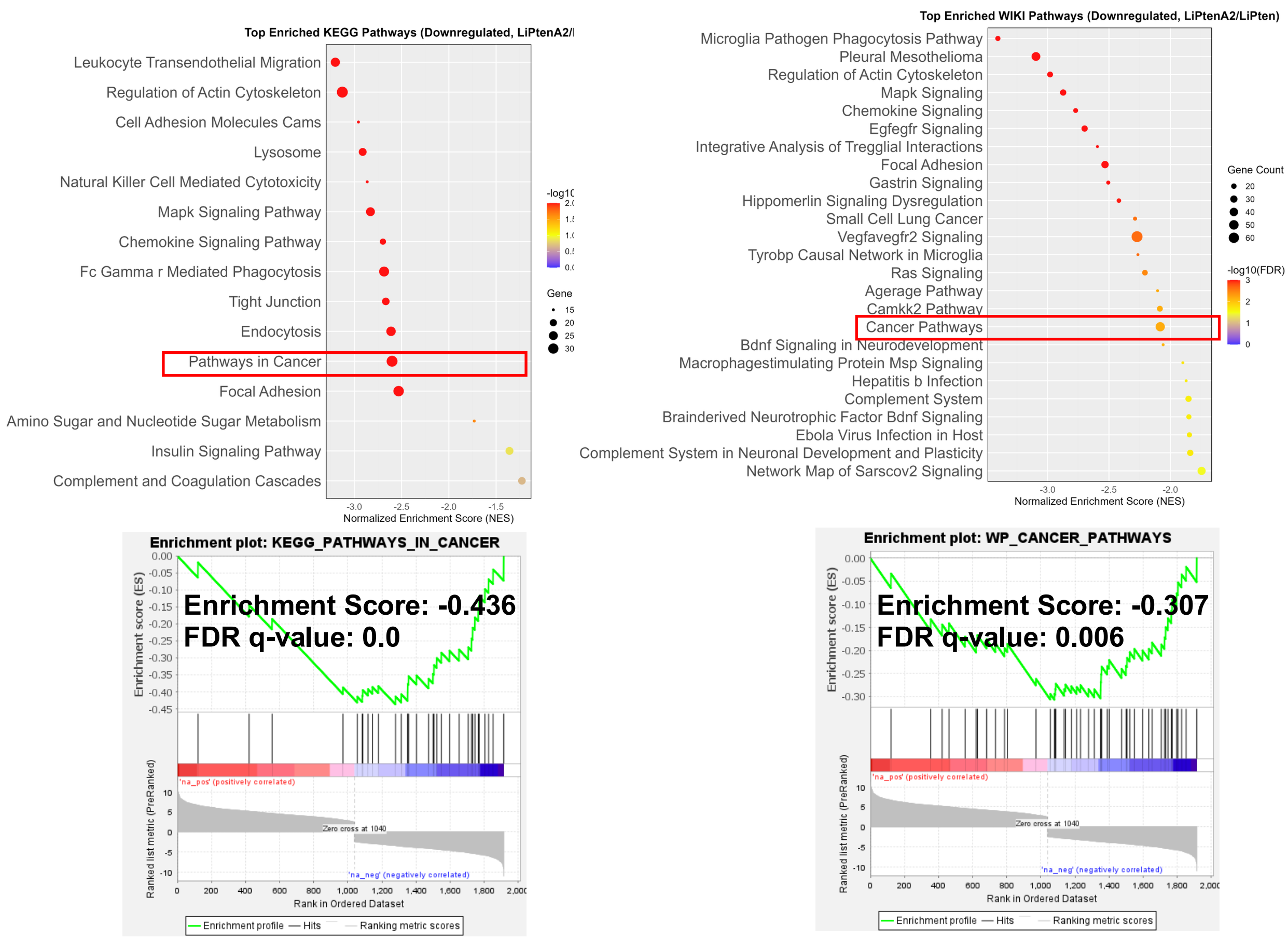


**Supplemental Figure 2. Loss of AKT2 rescues oncogenesis in livers lacking PTEN. (A)** PCR genotyping of tail DNA samples verified PTEN and AKT status in the respective mouse models. **(B)** Top downregulated KEGG and Wiki Pathways identified by proteomics in 12-month-old LiPtenA2 vs LiPten mice. Top, bubble plot highlighting cancer-related pathways. Bottom, enrichment plots of selected cancer pathways.

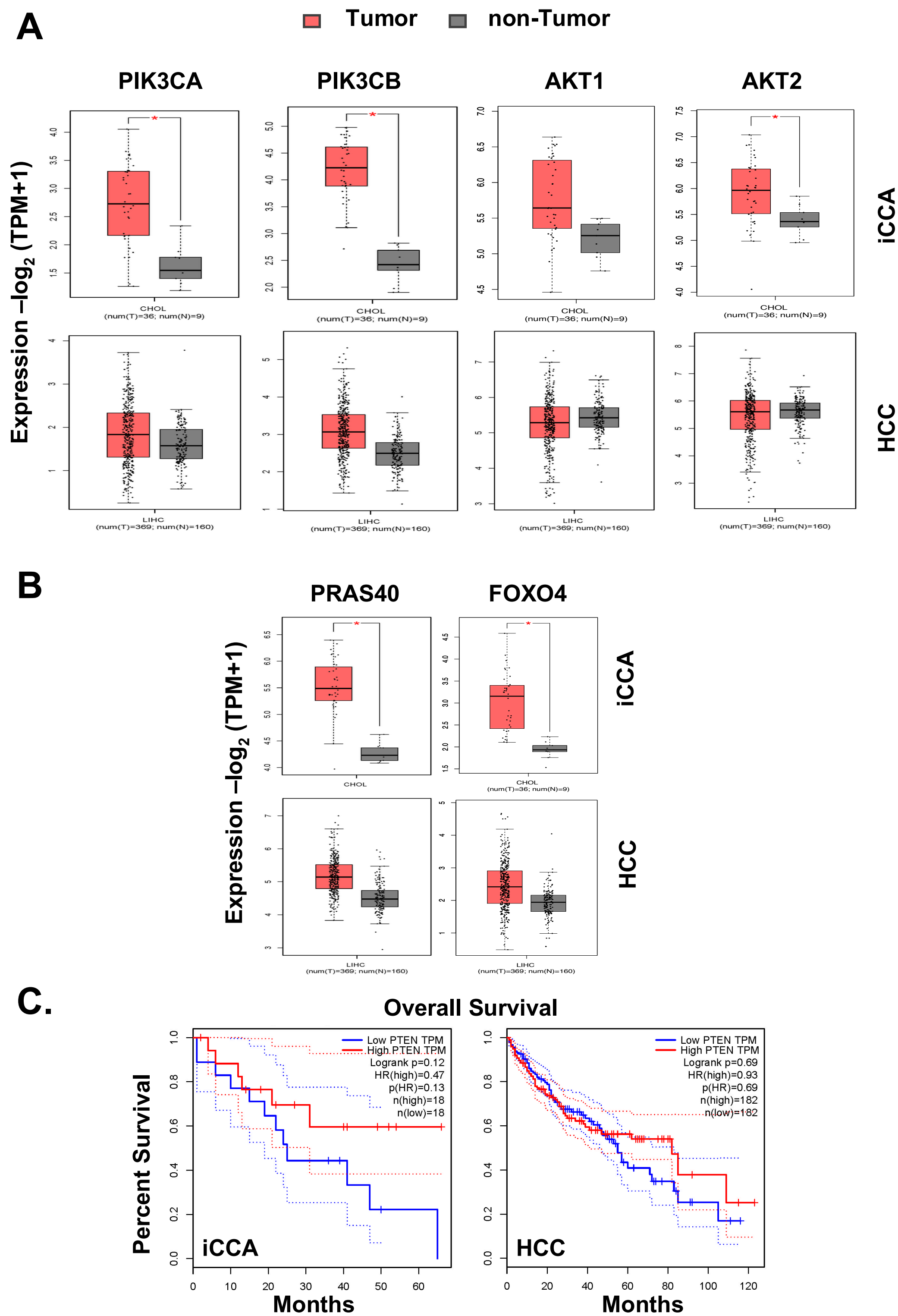


**Supplemental Figure 3. PI3K/AKT signaling pathway is correlated with CCA but not HCC.** Analysis of TCGA iCCA (n=36) and HCC samples (n=364) shows upregulation of PI3K and AKT isoforms **(A)** and AKT targets **(B)** mRNA expression in tumor vs. non-tumor samples in CCA but not HCC. **(C)** Overall survival analysis based on PTEN expression (Red, PTEN high, Blue, PTEN low) in the same iCCA and HCC samples. All analysis performed using GEPIA. *p≤0.05.

A

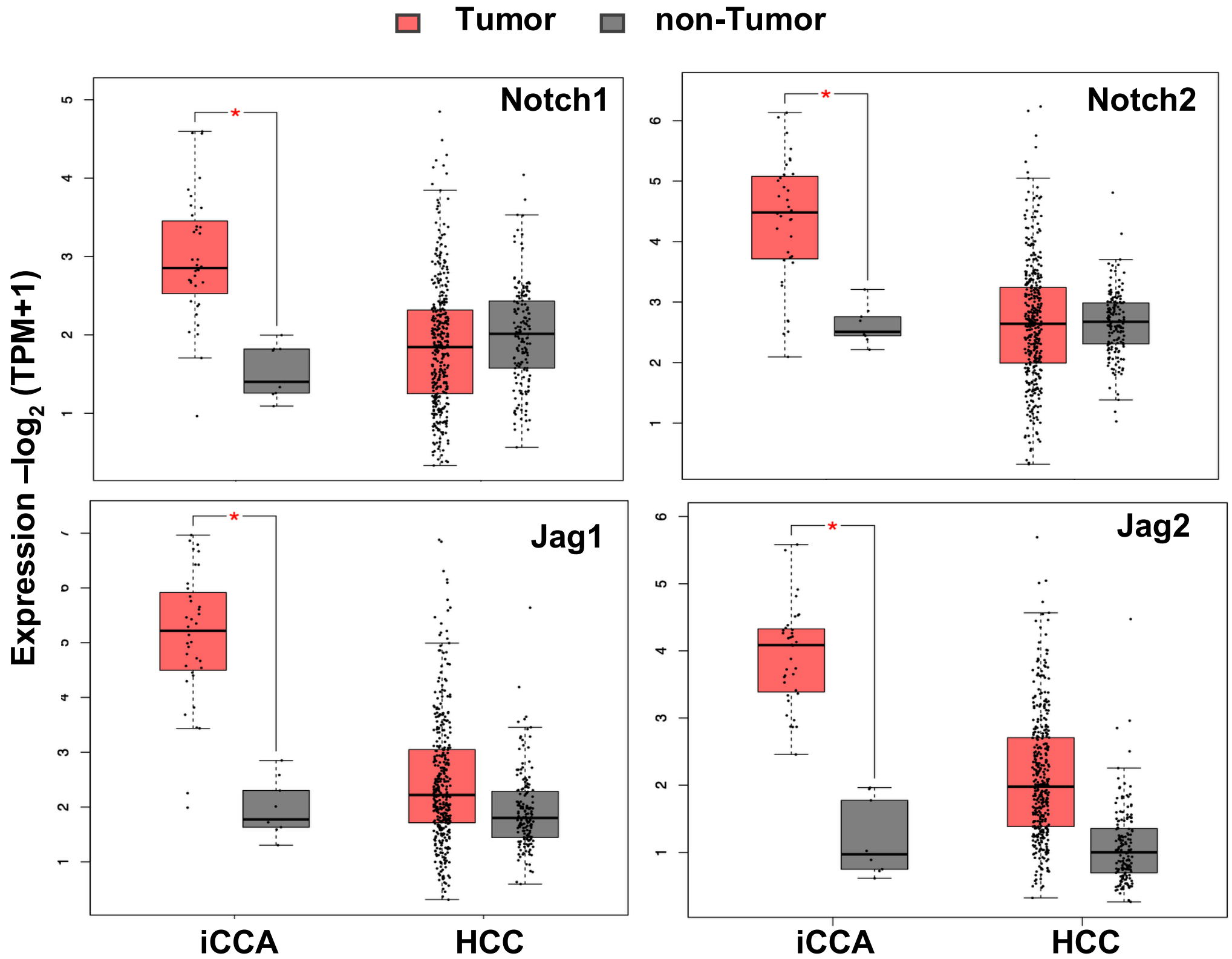


B

| Expression Correlation (R) * | | | | |
|---|---|---|---|---|
| | AKT1 | AKT2 | PIK3CA | PIK3CB |
| Notch1 | 0.54 | 0.39 | 0.57 | 0.49 |
| Notch2 | 0.42 | 0.45 | 0.68 | 0.71 |
| Notch3 | 0.35 | 0.33 | 0.55 | 0.48 |
| Notch4 | 0.21 | 0.11 | 0.2 | 0.13 |
| Jag1 | 0.29 | 0.32 | 0.53 | 0.45 |
| Jag2 | 0.33 | 0.29 | 0.44 | 0.51 |
| Hes1 | 0.35 | 0.2 | 0.42 | 0.41 |

***P<0.0001. expect Notch4-AKT2 p=0.03, Notch4-PIK3CA p=0.01**

**Supplemental Figure 4. Notch signaling is upregulated in CCA but not HCC and correlates well with PI3K/AKT signaling. (A)** Expression of Notch1 and 2 and Notch ligand Jag1&2 mRNA in TCGA iCCA (n=36) and HCC (n=364) samples using GEPIA. **(B)** Correlation analysis of Notch signal with that of PI3K/AKT. *P<0.0001 expect Notch4-AKT2 p=0.03, Notch4-PIK3CA p=0.01.

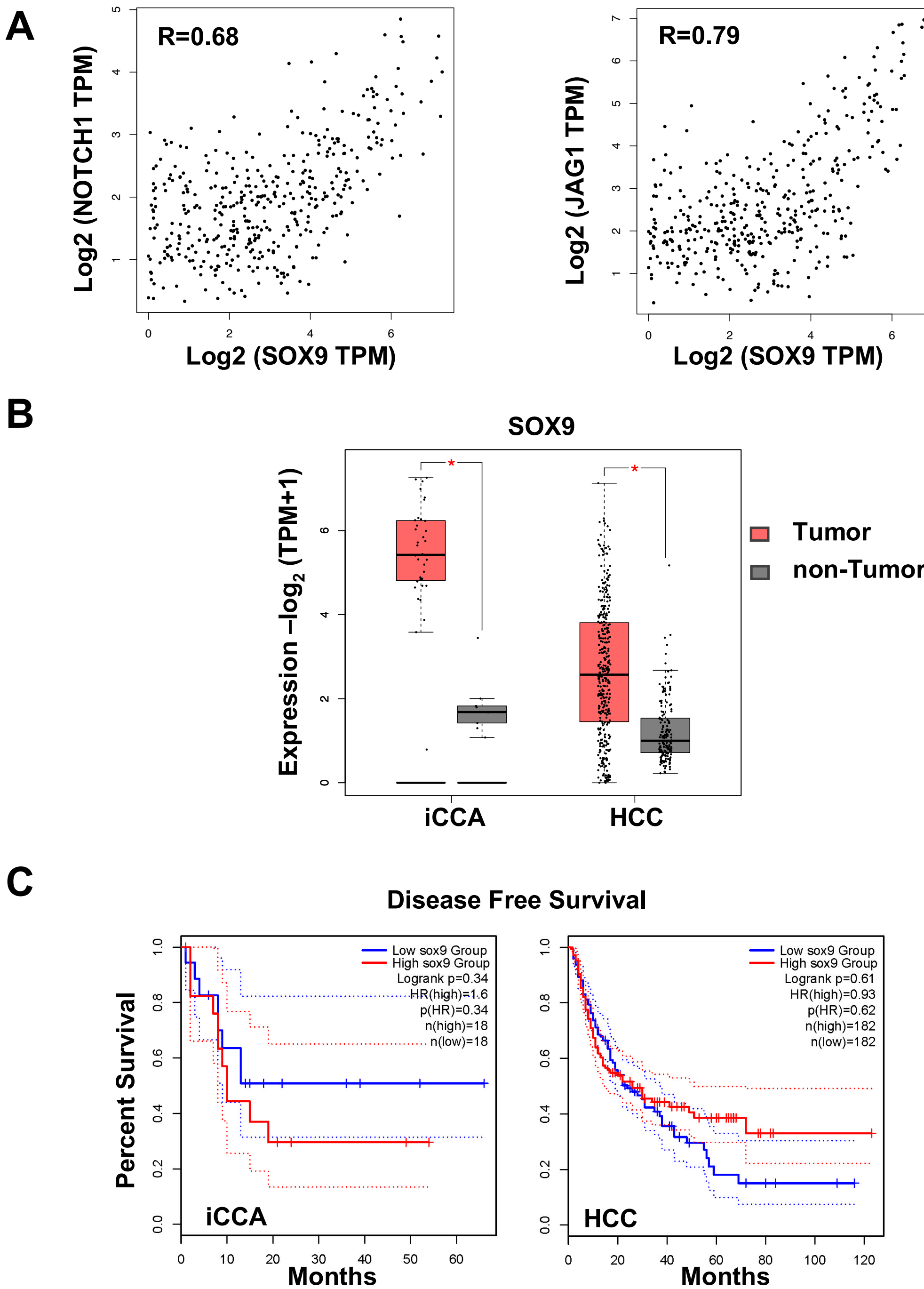


**Supplemental Figure 5. SOX9 expression in CCA/HCC and its correlation with NOTCH signal in patient samples from TCGA. (A)** Expression of NOTCH1 and JAG1 correlates well with that of SOX9 in liver cancer samples from TCGA. $p<0.01$. **(B)** Expression of SOX9 mRNA in the same TCGA samples. **(C)** Disease-free survival of SOX9 high (red) vs. low (blue). iCCA (n=36) vs. HCC patients (n=364) using GEPIA. $*p\leq0.05$.

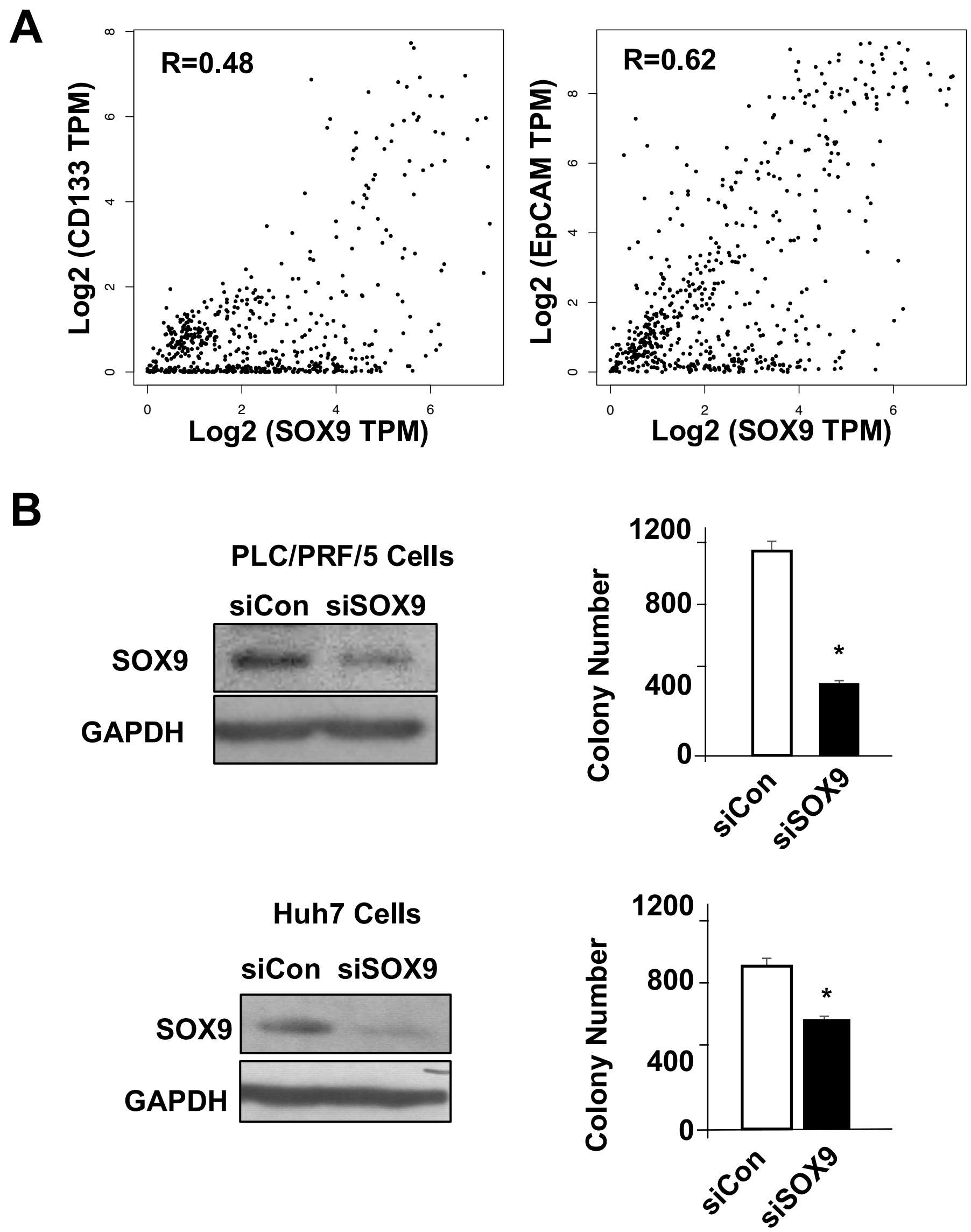


**Supplemental Fig 6. Correlation of SOX9 with stem cell markers and effects of its downregulation on colony formation. (A)** Expression of SOX9 correlates with that of CD133 and EpCAM in TCGA liver cancer samples. $p<0.01$. **(B)** Colony formation in PLC/PRF/5 cells and Huh7 cells with or without siSOX9 transfection. $*p \le 0.05$. n=3.

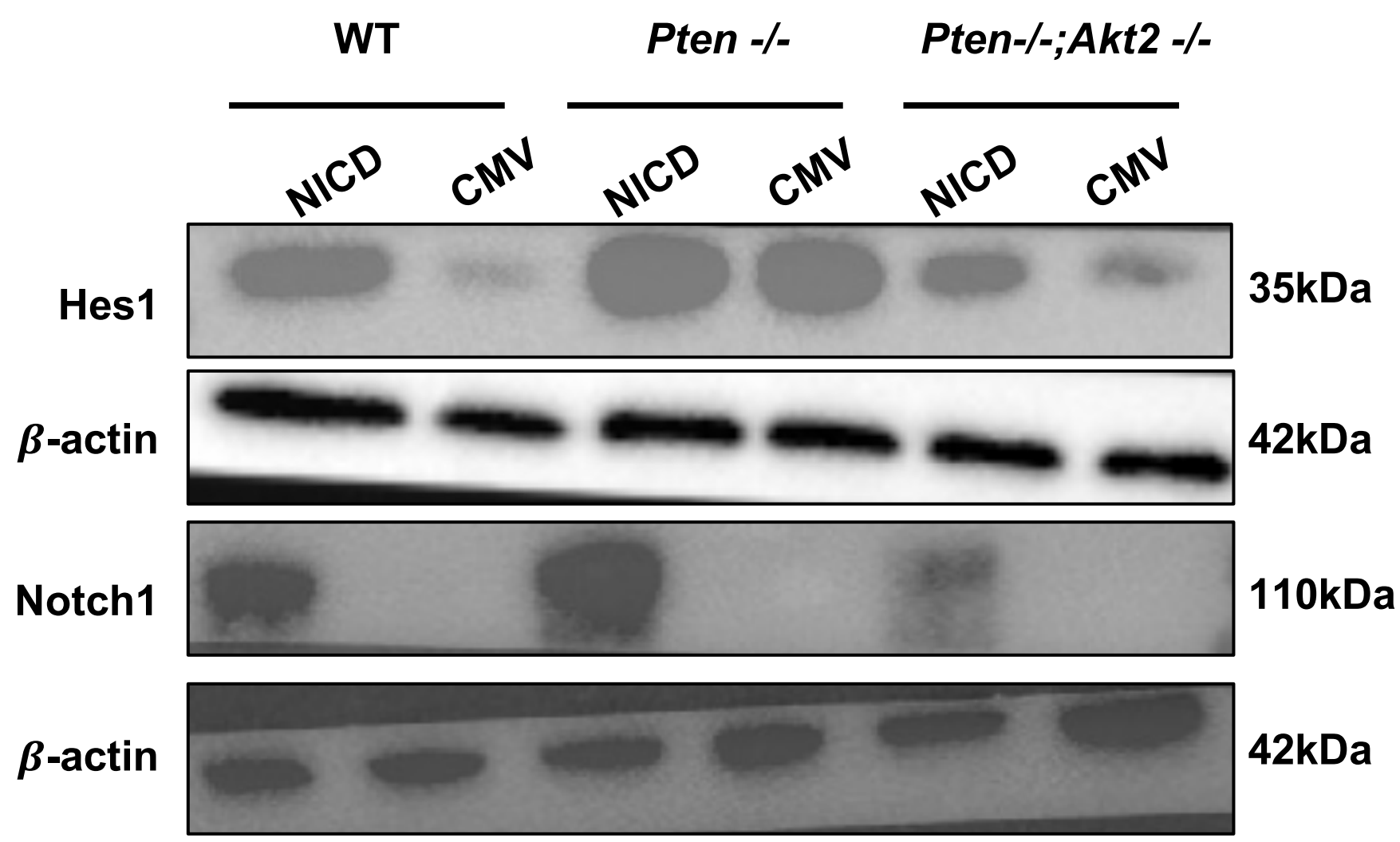


**Supplemental Fig 7. NICD introduction activates Notch signaling.** Immunoblotting shows NICD transfection significantly induces Notch signaling expression in WT, *Pten*$^{-/-}$ and *Pten*$^{-/-}$*; Akt2*$^{-/-}$ immortalized cells 24 hours after NICD transfection compared to CMV control. Blots are cropped for clarity.

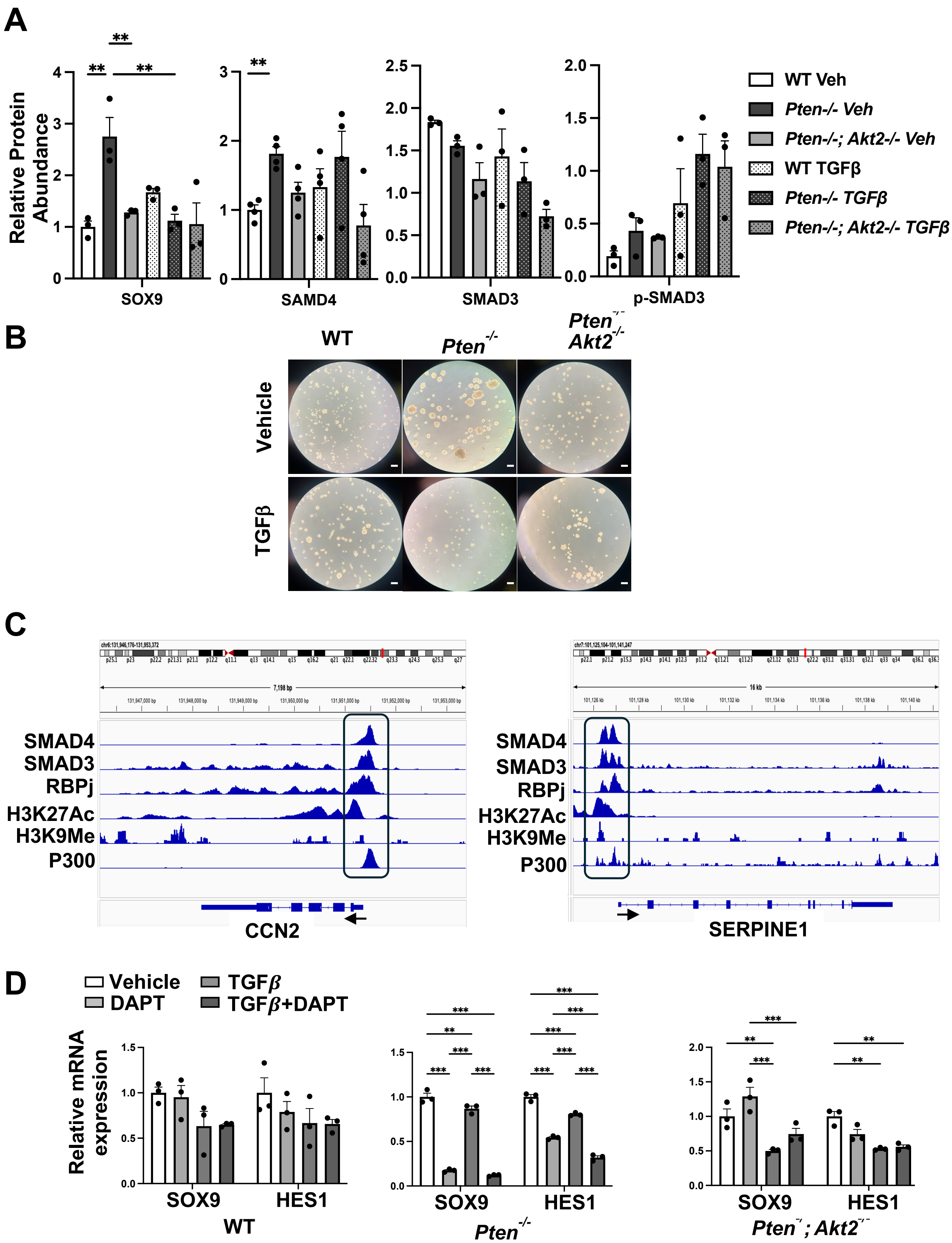
A
Relative Protein Abundance
SOX9
SAMD4
SMAD3
p-SMAD3
WT Veh
Pten-/- Veh
Pten-/-; Akt2-/- Veh
WT TGFβ
Pten-/- TGFβ
Pten-/-; Akt2-/- TGFβ
B
WT
Pten-/-
Pten-/- Akt2-/-
Vehicle
TGFβ
C
SMAD4
SMAD3
RBPj
H3K27Ac
H3K9Me
P300
CCN2
SERPINE1
D
Vehicle
DAPT
TGFβ
TGFβ+DAPT
Relative mRNA expression
SOX9
HES1
WT
Pten-/-
Pten-/-; Akt2-/-

**Supplemental Fig 8. PTEN loss induces different responses of liver tumor cells to TGFβ treatment. (A)** Quantification of relative protein abundance of immunoblotting showing expression of SOX9, SMAD4, SMAD3, and p-SMAD3 in WT, *Pten*$^{-/-}$ and *Pten*$^{-/-}$*; Akt2*$^{-/-}$ immortalized cells with or without TGFβ treatment. **(B)** Representative image showing TGFβ treatment inhibits sphere formation in *Pten*$^{-/-}$ cells but not WT cells and cells lacking AKT2 (*Pten*$^{-/-}$*; Akt2*$^{-/-}$) (300,000 cells seeded per 60mm dishes). **(C)** Promoter analysis of canonical TGFβ target genes. Encode ChIP-Seq data collected in HepG2 cells analyzed for promoters of CCN2 and SERPINE1, two canonical TGFβ target genes, showing SMAD3 and SMAD4 co-occupy the same active regulatory regions with RBPj. These peaks are also localized at the valley of H3K27Ac peaks, marking a transcriptional activation site. **(D)** qPCR analysis of SOX9 and Hes1 mRNA expression in WT, *Pten*$^{-/-}$ and *Pten*$^{-/-}$*; Akt2*$^{-/-}$ immortalized cells treated with TGFβ, DAPT or their combination under 48-hour sphere culture conditions.* $p< 0.05$, ** $p< 0.01$, *** $p< 0.001$.
